# Growth, Properties, and Theoretical Analysis of M_2_LiVO_4_ (M = Rb, Cs) Crystals: Two Potential Mid-Infrared Nonlinear Optical Materials

**DOI:** 10.1038/s41598-017-02117-0

**Published:** 2017-05-15

**Authors:** Guopeng Han, Ying Wang, Xin Su, Zhihua Yang, Shilie Pan

**Affiliations:** 1 Key Laboratory of Functional Materials and Devices for Special Environments, Xinjiang Technical Institute of Physics & Chemistry, Chinese Academy of Sciences, Xinjiang Key Laboratory of Electronic Information Materials and Devices, No. 40-1, South Beijing Road, Urumqi, 830011 China; 2 University of Chinese Academy of Sciences, Beijing, 100049 China

## Abstract

Mid-Infrared nonlinear optical (Mid-IR NLO) crystals with excellent performances play a particularly important role for applications in areas such as telecommunications, laser guidance, and explosives detection. However, the design and growth of high performance Mid-IR NLO crystals with large NLO efficiency and high laser-damage threshold (LDT) still face numerous fundamental challenge. In this study, two potential Mid-IR NLO materials, Rb_2_LiVO_4_ (RLVO) and Cs_2_LiVO_4_ (CLVO) with noncentrosymmetric structures (Orthorhombic, *Cmc*2_1_) were synthesized by high-temperature solution method. Thermal analysis and powder X-ray diffraction demonstrate that RLVO and CLVO melt congruently. Centimeter sized crystals of CLVO have been grown by the top-seeded solution growth method. RLVO and CLVO exhibit strong second harmonic generation (SHG) effects (about 4 and 5 times that of KH_2_PO_4_, respectively) with a phase-matching behavior at 1.064 μm, and a wide transparency range (0.33–6.0 μm for CLVO). More importantly, RLVO and CLVO possess a high LDT value (~28 × AgGaS_2_). In addition, the density functional theory (DFT) and dipole moments studies indicate that the VO_4_ anionic groups have a dominant contribution to the SHG effects in RLVO and CLVO. These results suggest that the title compounds are promising NLO candidate crystals applied in the Mid-IR region.

## Introduction

Materials with an enhanced nonlinear optical (NLO) response, in particular second-harmonic-generation (SHG), continue to attract much attention because of their great importance for optoelectronics^1, 2^. One of the efficient approach to realize tunable wavelength laser output is to convert the frequency from known lasers through NLO crystals. A good NLO crystal should possess some necessary properties for SHG applications, for example, wide transparency, large NLO coefficient, high laser-damage threshold (LDT), low absorption loss, and birefringently phase-matching^3^. Over the last three decades, many important inorganic NLO crystals have been discovered, such as *β*-BaB_2_O_4_ (BBO)^4^, LiB_3_O_5_ (LBO)^5^, CsLiB_6_O_10_ (CLBO)^6^, KH_2_PO_4_ (KDP)^7^, KTiOPO_4_ (KTP)^8^, KBe_2_BO_3_F_2_ (KBBF)^9^, LiNbO_3_ (LN)^10^. Although NLO materials used in ultraviolet (UV) and visible region can basically meet the requirements, strong interest also exists in the Mid-Infrared (IR) region to produce tunable coherent light source in the spectral range of 2–25 μm, including two important atmospheric transparent windows (3–5 and 8–14 μm)^11^. Currently, a few NLO crystals are commercially available in those spectral ranges. Benchmark materials such as ZnGeP_2_^12, 13^ and AgGaQ_2_ (Q = S, Se)^14, 15^ are the technologically mature IR NLO crystals with large NLO coefficient, low absorption coefficient, wide transparency range. However, these crystals suffer from two main drawbacks: (1) relatively narrow band gaps (*E*_g_) seriously limit the LDT; (2) difficulty in growing large crystal with high-quality seriously hinders practical applications. With the increasing demands of military and other civil applications, the exploration of next generation high performance Mid-IR NLO crystals has become the research focus of IR laser technology.

For designing high performance Mid-IR NLO materials, it is particularly difficult to screen new materials that simultaneously possess high NLO efficiency and LDT. General knowledge indicates that NLO materials with high LDT usually correspond to the large energy *E*_g_ value, whereas compounds with large *E*_g_ value often exhibit small NLO coefficients^16^. To search for new Mid-IR NLO crystals with excellent performance, it is interesting to note that chalcogenides are promising candidates because they usually have a wide transparency range and high NLO efficiency^17, 18^. Unfortunately, chalcogen atoms are polarized much more easily than oxygen, which also results in a smaller *E*_g_ value. In recent research, many studies pay special attention to halides, iodates, molybdates, and vanadates since they normally exhibit large *E*_g_ and diverse structural motifs so that it is possible to find materials with a subtle balance between large NLO efficiency and high LDT. A series of potential Mid-IR NLO crystals have been achieved, such as Pb_17_O_8_Cl_18_^19^, *α*-AgI_3_O_8_ and *β*-AgI_3_O_8_^20^, RbIO_3_^21^, Na_2_Te_3_Mo_3_O_16_^22^, BaTeMo_2_O_9_^23^, LiNa_5_Mo_9_O_30_^24^, K_3_V_5_O_14_^25, 26^ and YCa_9_(VO_4_)_7_^27^. After a lot of screening and system performance evaluation, we think that Rb_2_LiVO_4_ (RLVO) and Cs_2_LiVO_4_ (CLVO) are good Mid-IR NLO materials.

In this work, polycrystalline samples of RLVO and CLVO were prepared by standard solid state techniques. The crystal structure, physical and chemical properties of these two materials were studied. The thermal gravimetric analysis (TGA) and differential scanning calorimetry (DSC) measurements indicate that both RLVO and CLVO melt congruently. Single crystals of RLVO and CLVO were grown using the high temperature solution method, and centimeter-size crystals of CLVO were grown by the top-seeded solution growth (TSSG) method. The transmittance spectrum measurement indicates that CLVO has a wide transmission window range from 0.33 to 6.0 μm, which covers an important atmospheric transparent domain (3–5 μm). Remarkably, the reported materials show relatively large *E*_g_ values (3.8 eV for RLVO and 3.7 eV for CLVO) and high LDT (about 28 × AgGaS_2_ for RLVO and CLVO) among the known Mid-IR NLO crystals^17, 19, 21, 28^. The SHG measurements indicate that RLVO and CLVO have strong SHG efficiency of about 4 and 5 times that of KDP, respectively. These results show that the reported materials are two potential Mid-IR NLO candidate crystals with promising application in high-energy laser systems.

Besides, on the basis of these experimental results, the dipole moments calculation and first-principles calculations on the title compounds were performed to analyze the structure-property relationships.

## Results

**Figure 1:**
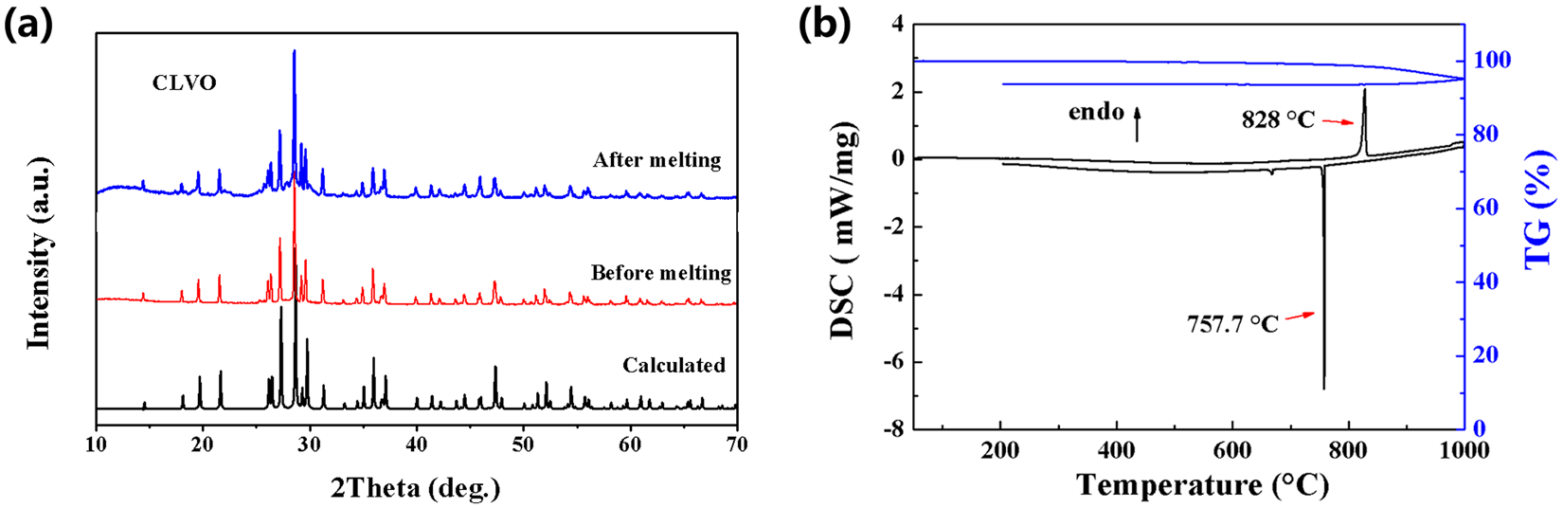
(**a**) Calculated and experimental PXRD for CLVO. (**b**) The TGA-DSC data for CLVO.

Polycrystalline samples of RLVO and CLVO were prepared by conventional solid-state reaction (see the Experimental Section). The powder X-ray diffraction (PXRD) patterns of the as-synthesized samples are shown good agreement with the calculated ones derived from the single-crystal data (Fig. 1a). The thermal behaviors of RLVO and CLVO were measured by TGA and DSC at a range of 50 to 1000 °C. For CLVO, the TGA curve demonstrates that it has no obvious weight loss up to 900 °C, and only one clear endothermic peak at 828 °C is observed in the heating curve of DSC, which was confirmed to be the melting point (Fig. 1b). In addition, powder samples of CLVO were heated to 850 °C to melt completely, then cooled to room temperature at a rate of 2 °C/h. Analysis of the PXRD pattern of the solidified melt indicates that the solid product is in good agreement with that of the initial powder (Fig. 1a). RLVO shows similar experimental results (Supplementary Figure 1). These results indicate that both RLVO and CLVO melt congruently, which imply that the two crystals can be grown from its stoichiometric melt.

**Figure 2:**
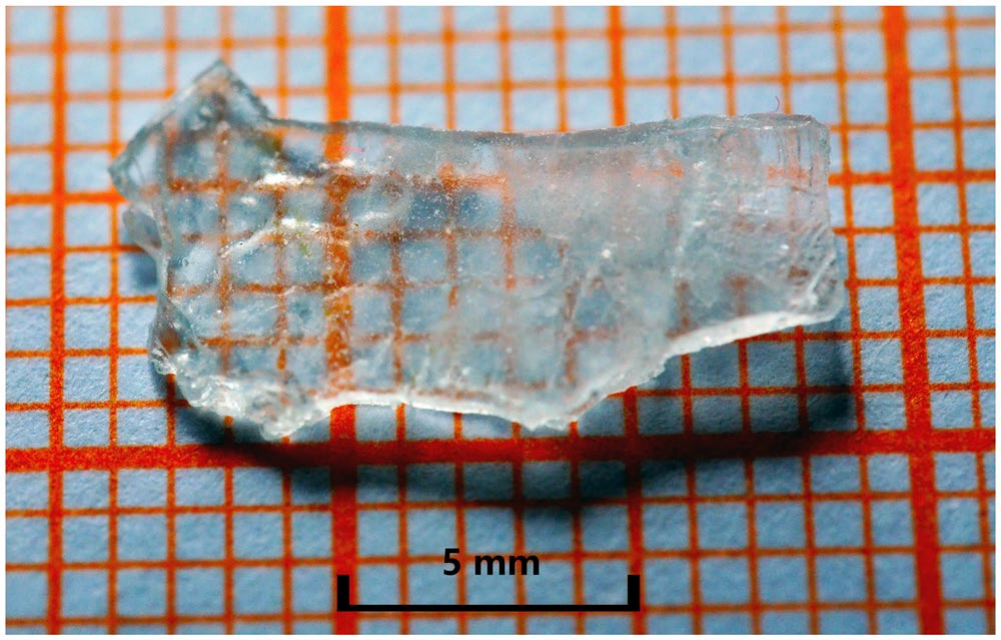
Photograph of the as-grown single crystal of CLVO.

Single crystal of CLVO was grown by TSSG method. Although CLVO melts congruently and can be grown from stoichiometric melt, the molar ratio *M*_Li2CO3_:*M*_Cs2CO3_:*M*_V2O5_ = 1:2.5:1 has been used to offset the impact of Cs_2_O evaporation in the high temperature. The phase purity of the obtained crystals through spontaneous crystallization was verified by PXRD. In the process of crystal growth, we found that the suitable cooling rate is essential for growing large transparent crystals. Under the cooling rate of 5 °C/day, the quality of as-grown crystals was poor and there were many millimeter-sized crystals on the solution surface. When the cooling rate reduced to 0.5–1 °C/day, the quality of crystals was evidently improved with fewer inclusions. It can be seen from Fig. 2 that the CLVO crystal with dimensions of about 13 × 7 × 2 mm^3^ was obtained. It is worth noting that we did not find unstable growth, such as byproducts which have often been observed for MgTeMoO_6_^29^. This indicates that large, high quality CLVO single crystal can be obtained more readily compared with MgTeMoO_6_.

**Figure 3:**
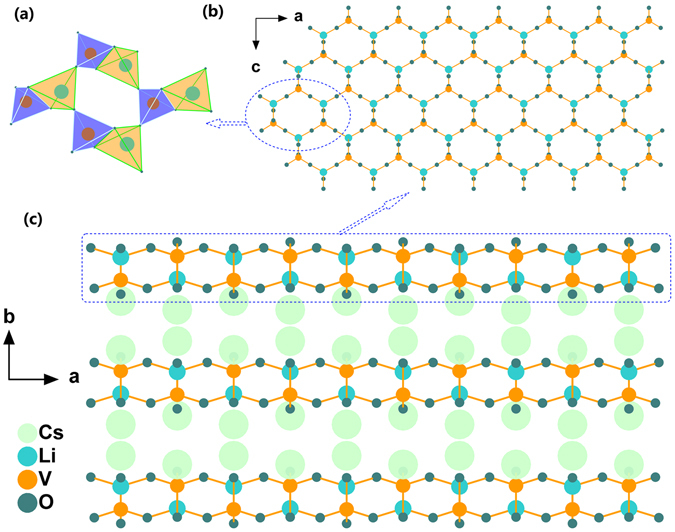
Crystal structure of CLVO. (**a**) Coordination environment of the LiO_4_ and VO_4_ tetrahedra; (**b**) The 2D [LiVO_4_]_∞_ layers viewing along the *b* axis; (**c**) The 3D crystal structure of CLVO. (LiO_4_ tetrahedra: yellow; VO_4_ tetrahedra, purple).

Single crystal XRD analysis reveals that RLVO and CLVO are isotypic, hence only the structure of CLVO will be discussed in detail, as a representation. CLVO crystallizes in the NCS orthorhombic polar space group *Cmc*2_1_, which was firstly reported by L. H. Brixner and C. M. Foris in 1989^30^. The relevant details of the data collections and evaluations are listed in Supplementary Table 1. In the asymmetric unit, there are two Cs atoms, one Li atom, one V atom and three O atoms. The V atom is tetrahedrally coordinated by four O atoms, forming a VO_4_ tetrahedron with V-O bond lengths ranging from 1.712 to 1.716 Å. The Li atom is bonded to four O atoms, forming a LiO_4_ tetrahedron with Li-O distances ranging from 1.884 to 2.137 Å. The LiO_4_ and VO_4_ tetrahedra link with each other by edge-sharing to form the [LiVO_6_] units, and the [LiVO_6_] units are further linked with each other by corner-sharing to yield two dimensional (2D) [LiVO_4_]_∞_ layers (Fig. 3a,b). The 2D [LiVO_4_]_∞_ layers are separated by the Cs^+^ cations to maintain charge balance and stack along the *b* axis (Fig. 3c).

The Cs1 and Cs2 atoms are connected by eight O atoms, forming CsO_8_ polyhedron with the Cs-O bond lengths ranging from 3.043 to 3.597 Å in CLVO, while the Rb atoms in RLVO possess two types of coordination environments, that is, Rb1O_10_ or Rb2O_8_. The Rb-O bond lengths in the Rb1O_10_ and Rb2O_8_ polyhedra range from 2.857 to 3.521 Å, and 2.856 to 3.240 Å, respectively. Based on bond valence calculations^31, 32^, the bond valence sums (BVS) indicate that all of atoms are in their normal oxidation states. (Supplementary Table 2).

IR spectroscopy was used to probe the coordination geometry of the vanadium cation in the title compounds. The IR spectra of title compounds showing the areas of interest are displayed in Supplementary Figure 2. The IR spectrum of the investigated RLVO and CLVO shows absorption bands ranging from 449 to 950 cm^−1^, which can be attributed to the stretching and bending vibrations of the V-O and O-V-O groups. The assignments are consistent with previous reports^33–35^.

**Figure 4:**
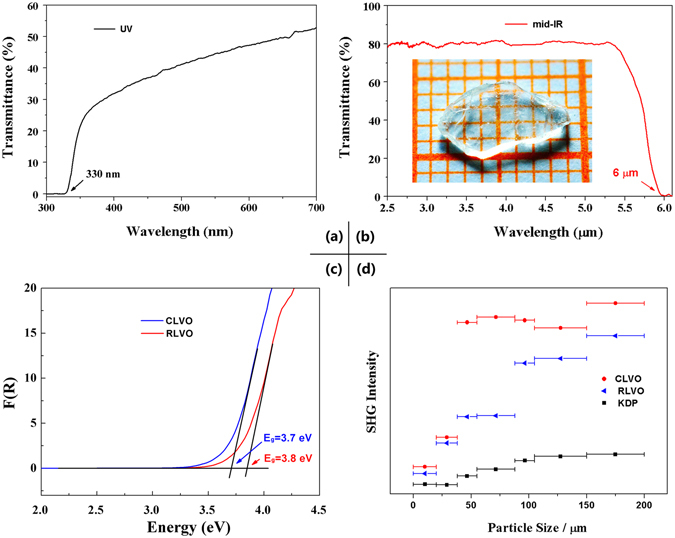
Linear and nonlinear optical properties of RLVO and CLVO. (**a**) The UV–vis–NIR and (**b**) Mid-IR transmittance spectrum on single crystal plate of CLVO, (**c**) UV-vis- NIR diffuse reflectance spectroscopy plots for RLVO and CLVO, (**d**) Phase-matching curves for RLVO and CLVO at 1064 nm.

To determine the transparency range of CLVO, the UV-vis-NIR and Mid-IR transmittance spectrum were collected on a 1 mm thick single crystal plate (without further polish). The results indicate that CLVO exhibits a very wide transmission range from 0.33 to 6.0 μm (Fig. 4a,b), compared to previously reported quaternary oxide crystals such as MgTeMoO_6_ (0.36–5.2 μm)^29^, Cs_2_TeMo_3_O_12_ (0.430–5.38 μm)^36^, Na_2_Te_3_Mo_3_O_16_ (0.42–5.4 μm)^22, 37^, LiNa_5_Mo_9_O_30_ (0.357–5.26 μm)^24^, *β*-BaTeMo_2_O_9_ (0.5–5.0 μm)^38^, and Na_2_TeW_2_O_9_ (0.36–5.0 μm)^39^. To evaluate the frequency doubling capabilities of RLVO and CLVO, SHG measurements were performed under a Q-switched Nd:YVO_4_ laser (*λ* = 1064 nm) according to the Kurtz-Perry method^40^. By measuring the SHG response as a function of particle size (ranging from 20 to 200 μm), the SHG intensities and phase-matching capability can be estimated. The results indicate that the title compounds exhibit strong SHG conversion efficiencies (*η* ^sample^/*η* ^KDP^) of about 4 (RLVO), 5 (CLVO) times that of the benchmark KDP in the particle size of 150–200 μm with a phase-matching behavior (Fig. 4d). In addition, UV-vis-NIR diffuse reflectance spectra for RLVO and CLVO in the region of 190–2600 nm were collected. As shown in Fig. 4c, the experimental *E*_g_ value of RLVO and CLVO is 3.8 and 3.7 eV, respectively (Fig. 4c). It is known that a high LDT in an NLO crystal usually corresponds to the large energy *E*_g_^41^, therefore, it is expected that these two crystals exhibit high LDT. In order to further verify these, a pulse laser was used to preliminarily assess the LDT of the compounds with AgGaS_2_ as the reference. The results indicate that both RLVO and CLVO have a high LDT (about 136 MWcm^−2^), which are about 28 times greater than that of AgGaS_2_ (4.8 MWcm^−2^). Remarkably, the reported materials show a relatively large *E*_g_ among the known Mid-IR NLO crystals with high LDT *i*.*e*., Pb_17_O_8_Cl_18_^19^ (3.44 eV and 12.8 × AgGaS_2_), RbIO_3_ (4.0 eV and about 20 × AgGaS_2_)^21^, Na_2_BaSnS_4_ (3.27 eV and about 5 × AgGaS_2_)^17^ and Na_2_ZnGe_2_S_6_ (3.25 eV and 6 × AgGaS_2_)^28^, which implies that the reported materials may be promising for application in high-energy laser systems. For comparison, although borate materials, such as *β*-BaB_2_O_4_ (BBO)^4^, LiB_3_O_5_ (LBO)^5^, possess a high LDT, they also show low optical transmittance in the Mid-IR (3–5 μm) range due to intrinsic vibration absorptions.

For the purpose of discussion, the structural building units and the whole structures of title compounds are illustrated in Fig. 3. To gain further insight into the electronic structure and optical properties of title compounds, electronic band structures were also calculated using CASTEP^42^. As shown in Supplementary Figure 3, RLVO and CLVO are found to be indirect band gap crystals (4.11 eV and 4.15 eV) since valence band (VB) maximum and conduction band (CB) minimum are located at different points. The difference of the band gap of two compounds mainly originates from the different atomic coordination around the oxygen anions.

**Figure 5:**
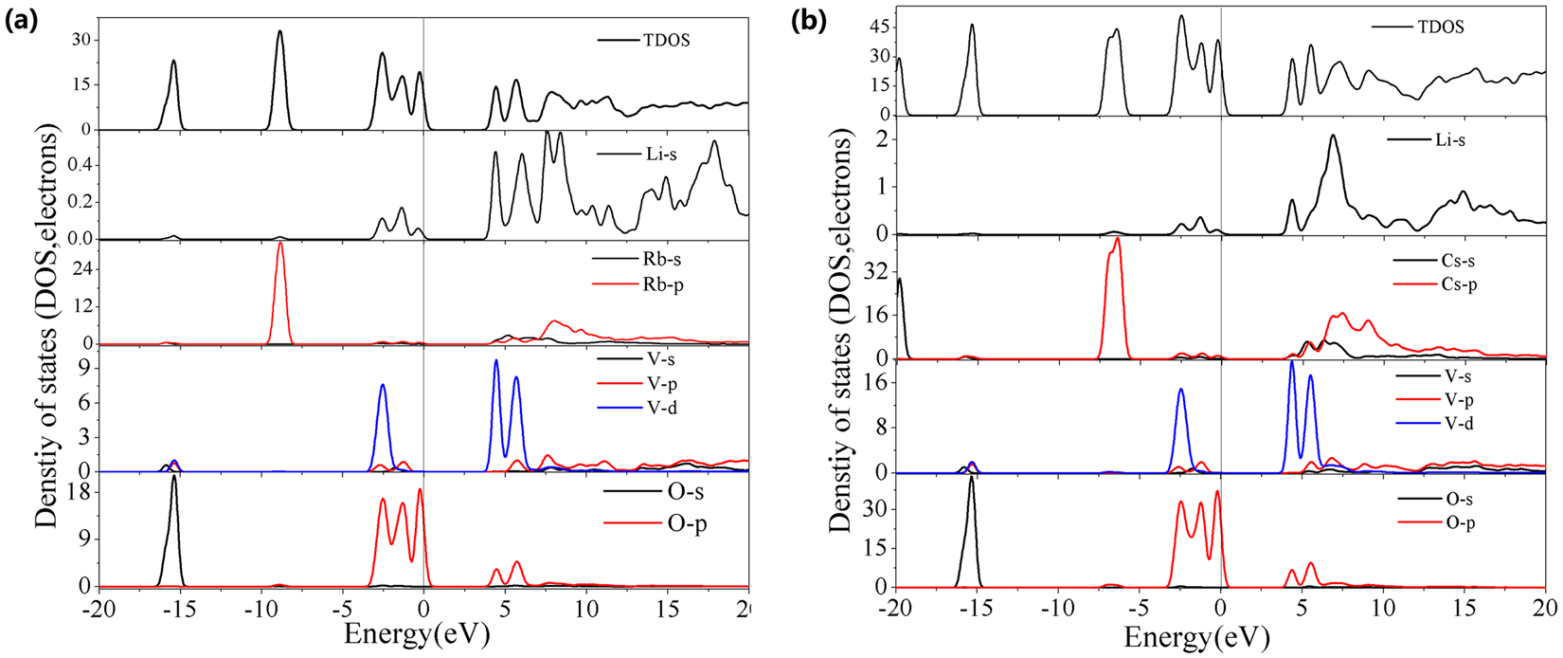
Partial density of states (PDOS) and total density of states (TDOS) of RLVO (**a**) and CLVO (**b**). The Fermi level is set as E = 0 eV.

The total density of states (TDOS) shows an overall orbital mixing of the four elements over the entire energy range as shown in Fig. 5. Obviously, the TDOS can be divided into several energy regions: 1) The lowest-energy region around −20 eV contributes from the 4*s* and 5*s* states of the Rb and Cs atoms, for RLVO and CLVO, respectively. 2) The region between −16.5 and −14.5 eV mostly displays contributions from the *s*, *p* and *d* states of the central V atom and the *p* orbitals of the surrounding four O atoms with some mixing of Rb/Cs *s* states. 3) The region between −8 and −10 eV includes strong contributions from the *p* states of the central Rb atom and the *p* orbitals of the O atoms with some mixing of Li *s* states in RLVO. Similarly, the region between −7.5 and −5.5 eV includes strong contributions from the *p* states of the central Cs atom and the *p* orbitals of O atoms with some mixing of Li *s* states in CLVO. 4) The region between −3.5 eV and the Fermi level is mostly dominated by the *p* states of O atoms with some contributions from the *p* states of both Rb/Cs and V. 5) The bottom of the conduction bands is mainly composed of 2*p* states of O, 3*d* states of V and orbitals of cations. Since the optical properties relate with the electron transition from the top of VB to the bottom of CB nearby the Fermi level^43^, one can deem that the VO_4_ anionic groups have a dominant contribution to the SHG response for these title compounds, and the contribution of cations cannot be neglected especially for rubidium and cesium whose orbitals have overlap with the V-O atoms.

The calculations of linear optical properties described in terms of the complex dielectric function *ε*(*ω*) = *ε*_1_(*ω*) + i*ε*_2_(*ω*) were made. The imaginary part of the dielectric function can be calculated with from matrix elements which describe the electronic transitions between the ground state and the excited states in the crystal considered. The imaginary part of the dielectric function *ε*_2_(*ω*) was given by the following equation^44^:

1 $${\rm{I}}{\rm{m}}[{\varepsilon }_{{\rm{i}}{\rm{j}}}(\omega )]=\frac{{e}^{2}}{\pi {m}^{2}\hslash }\sum _{{\rm{m}}{\rm{n}}}\int dk\frac{{f}_{nm}{P}_{nm}^{i}{P}_{mn}^{j}}{{\omega }_{nm}^{2}}\delta [{\omega }_{nm}-\omega ]$$

where *f*_nm_ = *f*_n_ − *f*_m_, and *f*_n_, *f*_m_ are Fermi factors. The real part of the dielectric function is obtained by the Kramers–Kronig transform^45^. The static and dynamic second-order nonlinear susceptibilities *χ*_abc_ (*ω*, *ω*, *ω*) were calculated based on the so-called length-gauge formalism by Aversa and Sipe^46, 47^. It is known that the second order susceptibility *χ* ^(2)^ is a double of the SHG coefficient *d*_ij_. According to the Kleimman symmetry relation^48–50^, there are two possible SHG coefficients *d*_31_ = *d*_15_, *d*_32_ = *d*_24_ for title compounds with *mm*2 point group symmetry. The calculated *d*_15_ of RLVO and CLVO are about 7.2 and 7.4 KDP(*d*_36_), respectively. The calculated SHG coefficient is essentially in agreement with the experimental values.

**Figure 6:**
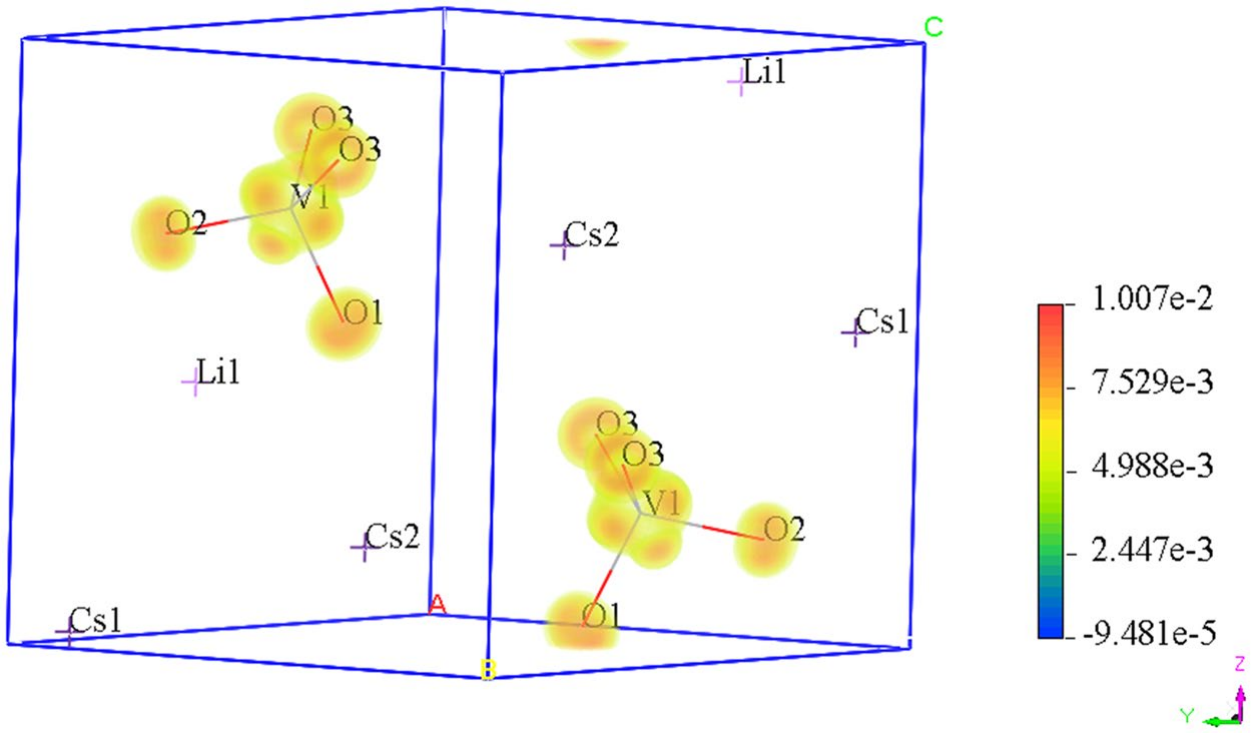
The SHG-density of the virtual-electron process of the largest SHG tensors of CLVO.

Furthermore, the density of SHG effect for RLVO and CLVO was calculated to further clarify the validity of the origin of SHG. As shown in Fig. 6, the SHG-density of CLVO mainly distributes around the VO_4_ anionic groups, that is to say, the VO_4_ anionic groups have a dominant contribution to the SHG coefficients. These results are in agreement with the largest orbital contributions near the Fermi level from the DOS and PDOS. Similar conclusions can also be obtained for RLVO (Supplementary Figure 4).

## Discussion

**Figure 7:**
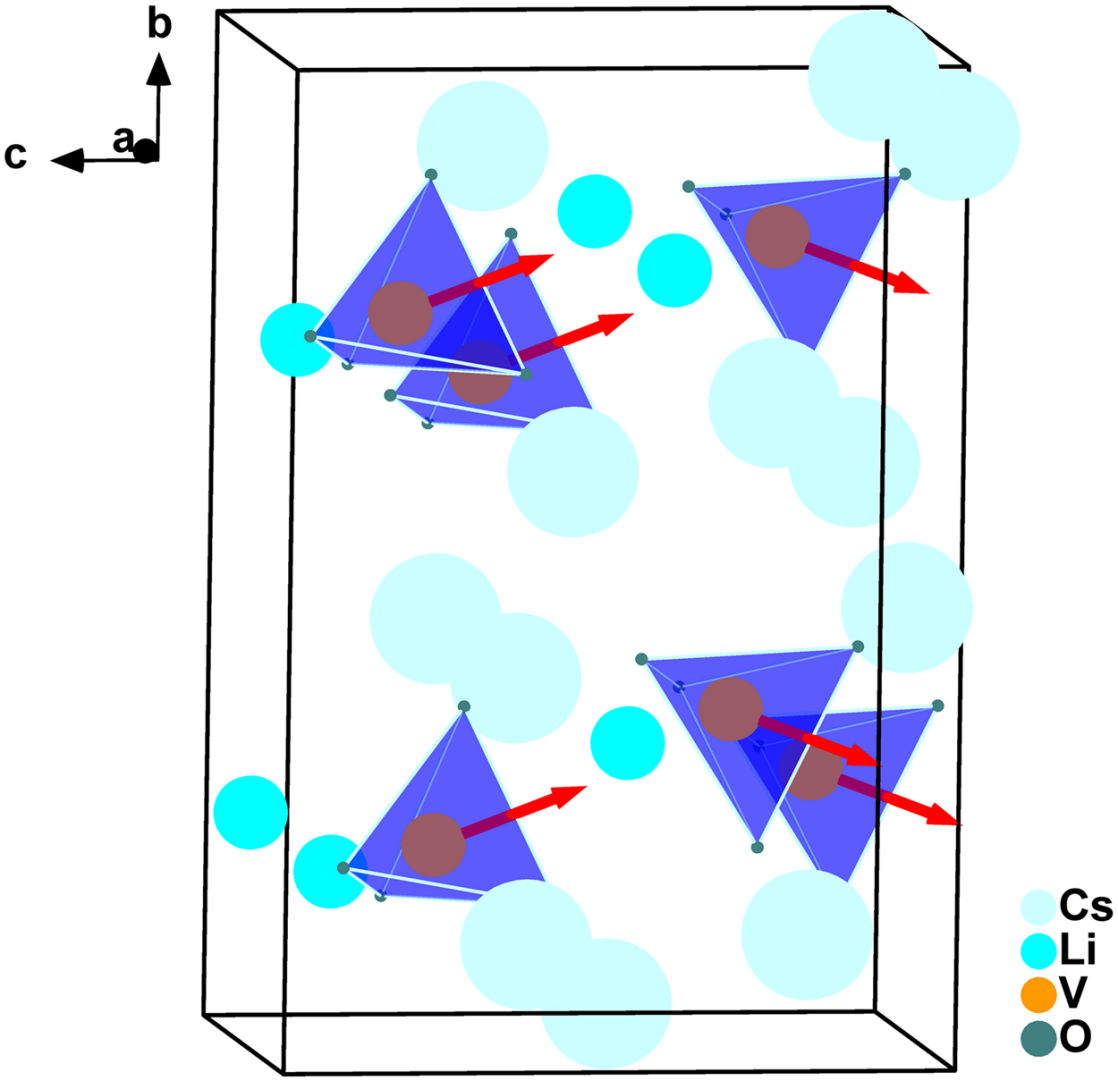
The direction of the dipole moments for the VO_4_ tetrahedra in the unit cell of CLVO. (The red arrows indicate the approximate directions of the dipole moments).

The optical properties of the title componds can also be elucidated from structural features. Since the SHG effect depends on not only the type of the anionic groups, but also the orientation of the anionic groups. The local dipole moments of the VO_4_ groups in title compounds were calculated by using a bond-valence approaching methodology^51^. The result reveals that the direction of the VO_4_ tetrahedra local dipole moments have two different orientations (Fig. 7, Supplementary Table 4, and Supplementary Figure 5), which lead to partial cancellation of the net dipole moments (In analogy to the PO_4_ tetrahedra in LiCs_2_PO_4_^52, 53^). The *b*-component of VO_4_ polarization cancels out completely in a unit cell, while the *c*-component of VO_4_ polarization constructively adds to a net value of 9.038 and 9.915 Debye. (Supplementary Table 4a). Besides, we should be careful when using dipole moments calculation to explain the origin of SHG response of RLVO and CLVO. Only anionic groups should be taken into account. As a counter example, the LiO_4_ tetrahedra have largest local dipole moments value (Supplementary Table 4b) while barely contribute to the SHG coefficients in first principles calculations.

In summary, two new Mid-IR NLO materials, RLVO and CLVO, were synthesized by solid state method. The structure of title compounds consists of the VO_4_ and LiO_4_ tetrahedra, forming 2D [LiVO_4_]_∞_ layers that are separated by the Rb^+^/Cs^+^ cations to maintain charge balance. A transparent CLVO crystal with sizes up to 13 × 7 × 2 mm^3^ was obtained by the TSSG method. A complete survey of linear and nonlinear optical properties for title compounds was demonstrated. The results indicate that the title compounds exhibit not only relatively large *E*_g_ (3.8 and 3.7 eV, respectively) and wide transparent region (0.33–6.0 μm for CLVO) among the known Mid-IR NLO crystals, but also achieve the suitable balance between large SHG response (4 and 5 that of KDP, respectively) and high LDT (about 28 × AgGaS_2_ for RLVO and CLVO). Besides, analysis of the SHG-density method and dipole moments studies reveal that the large SHG response of RLVO and CLVO mainly comes from the VO_4_ anionic groups. All these results suggest that RLVO and CLVO are attractive candidates for application in high-energy laser systems in Mid-IR region. This work also paves the way for exploiting Mid-IR NLO materials based on vanadates.

## Methods

### Synthesis

High-purity raw materials, Cs_2_CO_3_ (99%), Rb_2_CO_3_ (99%), Li_2_CO_3_ (99.95%), and V_2_O_5_ (99.9%), were used as received. Polycrystalline samples of RLVO and CLVO were prepared by the conventional high-temperature solid-state reaction techniques. A separate stoichiometric mixture of Cs_2_CO_3_-Rb_2_CO_3_, Li_2_CO_3_, and V_2_O_5_ were ground in an agate mortar and then heated progressively up to 620 °C for 6 days in air. In order to facilitate the completion of the reactions, some intermediate grindings are necessary. The purity of samples was checked by PXRD at room temperature as shown in Supplementary Figure 1. Small single crystals of RLVO and CLVO were grown through spontaneous crystallization from stoichiometric composition high-temperature melts.

### Crystal Growth

Single crystals of CLVO were grown by the TSSG method. The saturation temperature was determined by observing the growth or dissolution of the seed crystals when soaking in the melt. The seeds of CLVO were selected from the spontaneous crystallization. The molar ratio of raw materials is Li_2_CO_3_:Cs_2_CO_3_:V_2_O_5_ = 1:2.5:1. The prepared mixture was sintered at 620 °C for 48 h with several intermediate grindings. The well-mixed powder was put in the platinum crucible, which was placed in the middle of a vertical, programmable temperature furnace. Then, it was heated to 740 °C and held at this temperature for 10 h to ensure complete melting and homogeneity of the raw materials. A high-quality seed crystal was attached to a platinum rod and dipped into the solution at a temperature 3 °C above the saturation temperature. In the crystal growth, the temperature was cooled at a rate of 0.5–1 °C/day, and the seed rod was rotated at 5 rpm. When the growth was completed, the crystals were drawn out form the solution and cooled to room temperature at a rate of 10 °C/h.

### Material Characterization

PXRD analysis of RLVO and CLVO was performed at room temperature by a Bruker D2 PHASER X-ray diffractometer equipped with Cu Kα radiation (*λ* = 1.5418 Å). The 2*θ* range was 10–70° with a scan step width of 0.02° and a fixed counting time of 1 s per step. Single crystals of RLVO and CLVO were selected for the structure determination. Data were collected on a Bruker SMART APEX II CCD diffractometer using graphite-monochromatic Mo Kα radiation (*λ* = 0.71073 Å) at 296(2) K and integrated with the SAINT program^54^. The calculations were performed using the SHELXTL software package^55^. Crystallographic data for title compounds are reported in Supplementary Table 1, and the positional parameters, anisotropic displacement parameters, bond valence sums, interatomic distances, and angles are reported in Supplementary Tables 2 and 3, respectively.

Diffuse reflectance spectra for the polycrystalline samples was measured from 190 to 2600 nm using a SolidSpec-3700DUV spectrophotometer equipped with an integrating sphere attachment. The UV–vis–NIR and mid-IR transmittance spectra were measured on single-crystal plates with a thickness of 1 mm. The IR spectra of RLVO and CLVO in the range of 4000–400 cm^−1^ was measured on a Shimadzu IR Affinity-1 Fourier transform Infrared spectrometer, using the KBr-pellet technique. Thermal gravimetric analysis (TGA) and differential scanning calorimetry (DSC) were carried out using NETZSCH STA 449C thermal analyzer instrument in the range of 50–1000 °C with a heating rate of 10 °C·min^−1^. Second harmonic generation (SHG) testing was evaluated by using the Kurtz-Perry method^40^. The sample was pressed between glass slides in a 1-mm-thick aluminum cell, and then was irradiated by a pulsed Nd:YVO_4_ solid-state laser (λ = 1064 nm, 10 kHz, 10 ns). Since the SHG intensity depends strongly on the particle size of the sample, polycrystalline samples of RLVO and CLVO were sieved into a series of distinct size ranges of <20, 20–38, 38–55, 55–88, 88–105, 105–150 and 150–200 μm, respectively. The commercial KDP samples with the same particle size ranges were served as the references. Besides, the LDT values of powder compounds were measured under a Q-switch laser (1064 nm, 10 Hz, and 10 ns), and powder AgGaS_2_ sample was used as a reference in the same condition. The color change of the powder sample observed by optical microscope was adopted to determine the LDT when laser energy increased.

### Numerical Calculation Details

The electronic structures calculations were performed using a plane-wave basis set and pseudopotentials within density functional theory (DFT) implemented in the total-energy module CASTEP^42^. The exchange and correlation effects were treated by Perdew–Burke–Ernzerhof (PBE) in the generalized gradient approximation (GGA)^56^. The interactions between the ionic cores and the electrons were described by ulstrasoft pseudopotentials^57^. The following orbital electrons were treated as valence electrons: Rb 4*s* ^2^4*p* ^6^5*s* ^1^, Cs 5*s* ^2^5*p* ^6^6*s* ^1^, Li 1*s* ^1^, V 3*d* ^3^4*s* ^2^, and O 2*s* ^2^2*p* ^4^. The number of plane waves included in the basis was determined by a cutoff energy of 380 eV, and the numerical integration of the Brillouin zone was performed using a 6 × 3 × 4 Monkhorst-Pack scheme *k*-point grid sampling for M_2_LiVO_4_ (M = Rb, Cs). Our tests suggest that these computational parameters ensure good convergence in the present studies.

Based on the optimized geometries of the M_2_LiVO_4_ (M = Rb, Cs) crystals, the electronic band structures were calculated from the optical matrix transition elements between occupied and unoccupied states determined. The SHG coefficients were calculated by our improved calculation formula^58^ which has been successfully applied on a lot of NLO crystals such as KBe_2_BO_3_F_2_^59^.

## Electronic supplementary material

Supplementary information
