# Supplementary material for: Growth, Properties, and Theoretical Analysis of M2LiVO4 (M = Rb, Cs) Crystals: Two Potential Mid-Infrared Nonlinear Optical Materials
Source: Sci Rep. 2017 May 15;7:1901. doi: 10.1038/s41598-017-02117-0 (PMC5432528; doi:10.1038/s41598-017-02117-0)
Supplement: Supplementary file 1 — Supplementary information [file 41598_2017_2117_MOESM1_ESM.pdf]

Supplementary Information for:

Growth, Properties, and Theoretical Analysis of M_2LiVO_4 (M = Rb, Cs) Crystals: two Potential Mid-Infrared Nonlinear Optical Materials

Guopeng Han,[a,b] Ying Wang,[*,a] Xin Su,[a,b] Zhihua Yang,[a] and Shilie Pan[*,a]

[1]*Key Laboratory of Functional Materials and Devices for Special Environments, Xinjiang Technical Institute of Physics & Chemistry, Chinese Academy of Sciences, Xinjiang Key Laboratory of Electronic Information Materials and Devices, No. 40-1, South Beijing Road, Urumqi 830011, China*

[2]*University of Chinese Academy of Sciences, Beijing 100049, China*

*To whom correspondence should be addressed.

E-mail: wangying@ms.xjb.ac.cn, slpan@ms.xjb.ac.cn

Phone: (+86)991-3674558, Fax: (+86)991-3838957

CONTENTS

1. Crystal Data……………………………………….……….….....S3

2. PXRD Pattern and TG-DSC Data………………..………………S9

3. IR Spectrum………………………….……….………….....…..S10

4. Electron Band Structure………………………………….….......S11

5. The SHG Density…………………………….……………..…...S12

6. Dipole Moments Calculation……………………………….......S13

Supplementary Table 1. Crystal data and structure refinement for RLVO and CLVO.

Empirical formula	Rb_2LiVO_4	Cs_2LiVO_4
Temperature	296(2) K	296(2) K
Crystal system, space group	Orthorhombic, *Cmc*2(1)	Orthorhombic, *Cmc*2(1)
Unit cell dimensions	a = 5.836(6) Å	a = 6.002(12) Å
	b = 11.646(12) Å	b = 12.19(2) Å
	c = 7.879(8) Å	c = 8.203(16) Å
Volume	535.6(10) $Å^3$	600(2) $Å^3$
Z, Calculated density	4, 3.631 g cm^{-3}	4, 4.290 g cm^{-3}
Absorption coefficient	19.816 mm^{-1}	13.521 mm^{-1}
F(000)	528	672
Crystal size	0.117 mm × 0.115 mm × 0.09 mm	0.23 mm × 0.161 mm × 0.103 mm
Theta range for data collection	3.50 to 29.66°	3.78 to 27.64°
Limiting indices	$-5 \le h \le 8$, $-16 \le k \le 15$, $-10 \le l \le 9$	$-4 \le h \le 7$, $-13 \le k \le 15$, $-10 \le l \le 9$
Reflections collected / unique	1737 / 734 [R(int) = 0.0483]	1782 / 743 [R(int) = 0.0224]
Completeness to theta = 27.39	100.0 %	99.0 %
Data / restraints / parameters	743 / 1 / 46	743 / 1 / 46
Goodness-of-fit on F^2	0.830	1.094
Final R indices [$F_o^2>2\sigma(F_o^2)$] [α]	R_1 = 0.0265, wR_2 = 0.0484	R_1 = 0.0181, wR_2 = 0.0374
R indices (all data)[α]	R_1 = 0.0345, wR_2 = 0.0509	R_1 = 0.0184, wR_2 = 0.0375
Absolute structure parameter	0.007(16)	0.01(5)
Extinction coefficient	-	-
Largest diff. peak and hole	0.663 and -1.077 e $Å^{-3}$	0.519 and -1.011 e $Å^{-3}$

[α]$R_1 = \Sigma||F_o| - |F_c||/\Sigma|F_o|$ and $wR_2 = [\Sigma w(F_o^2 - F_c^2)^2/\Sigma wF_o^4]^{1/2}$ for $F_o^2 > 2\sigma(F_o^2)$

Supplementary Table 2. (a) Atomic coordinates (×10^4), equivalent isotropic displacement parameters (Å^2 × 10^3) for RLVO. U(eq) is defined as one third of the trace of the orthogonalized U_{ij} tensor.

Atoms	Wyck	*x*	*y*	*z*	*U*(eq)	BVS
Rb (1)	4*a*	0	1051(1)	9568(1)	21(1)	1.013
Rb (2)	4*a*	0	4381(1)	8121(1)	20(1)	0.989
Li(1)	4*a*	5000	2014(13)	7280(20)	21(3)	1.100
V(1)	4*a*	0	1905(1)	5156(2)	12(1)	5.238
O(1)	4*a*	0	3359(4)	4826(7)	20(1)	2.105
O(2)	8*b*	2375(6)	1467(3)	6219(7)	25(1)	2.065
O(3)	4*a*	0	1324(4)	3172(8)	18(1)	2.105

Supplementary Table 2. (b) Atomic coordinates (×10^4), equivalent isotropic displacement parameters (Å^2 × 10^3) for CLVO. U(eq) is defined as one third of the trace of the orthogonalized U_{ij} tensor.

Atoms	Wyck	*x*	*y*	*z*	*U*(eq)	BVS
Cs(1)	4*a*	0	685(1)	5210(1)	20(1)	0.975
Cs(2)	4*a*	0	3936(1)	6625(1)	19(1)	1.011
Li(1)	4*a*	5000	2984(12)	4340(15)	19(2)	1.012
V(1)	4*a*	0	3020(1)	2211(1)	12(1)	5.091
O(1)	4*a*	0	3637(4)	331(6)	17(1)	2.048
O(2)	4*a*	0	1643(4)	1785(6)	21(1)	2.133
O(3)	8*b*	2347(6)	3384(3)	3267(5)	24(1)	1.954

Supplementary Table 3. (a) Selected bond lengths (Å) and angles (°) for RLVO.

Li(1)-O(2)#1	1.858(9)	Rb(1)-O(2)#11	3.521(5)
Li(1)-O(2)	1.858(9)	Rb(2)-O(1)	2.856(6)
Li(1)-O(1)#2	2.053(17)	Rb(2)-O(1)#12	2.955(5)
Li(1)-O(3)#2	2.059(16)	Rb(2)-O(3)#8	3.032(3)
Rb(1)-O(3)#6	2.857(7)	Rb(2)-O(3)#2	3.032(3)
Rb(1)-O(3)#7	2.977(6)	Rb(2)-O(2)#2	3.047(5)
Rb(1)-O(1)#2	3.005(3)	Rb(2)-O(2)#11	3.047(5)
Rb(1)-O(1)#8	3.005(3)	Rb(2)-O(2)#14	3.240(5)
Rb(1)-O(2)#9	3.020(6)	Rb(2)-O(2)#13	3.240(5)
Rb(1)-O(2)	3.020(6)	V(1)-O(2)	1.698(4)
Rb(1)-O(2)#7	3.494(5)	V(1)-O(2)#9	1.698(4)
Rb(1)-O(2)#10	3.494(5)	V(1)-O(3)	1.704(6)
Rb(1)-O(2)#2	3.521(5)	V(1)-O(1)	1.713(5)
O(2)#1-Li(1)-O(2)	111.1(8)	O(3)#7-Rb(1)-O(2)#11	154.09(7)
O(2)#1-Li(1)-O(1)#2	111.5(6)	O(1)#2-Rb(1)-O(2)#11	102.11(12)
O(2)-Li(1)-O(1)#2	111.5(6)	O(1)#8-Rb(1)-O(2)#11	50.56(10)
O(2)#1-Li(1)-O(3)#2	118.4(5)	O(2)#9-Rb(1)-O(2)#11	89.50(8)
O(2)-Li(1)-O(3)#2	118.4(5)	O(2)-Rb(1)-O(2)#11	113.00(9)
O(1)#2-Li(1)-O(3)#2	82.3(6)	O(2)#7-Rb(1)-O(2)#11	112.26(12)
O(3)#6-Rb(1)-O(3)#7	118.10(15)	O(2)#10-Rb(1)-O(2)#11	136.38(17)
O(3)#6-Rb(1)-O(1)#2	84.68(10)	O(2)#2-Rb(1)-O(2)#11	51.58(13)
O(3)#7-Rb(1)-O(1)#2	103.74(9)	O(1)-Rb(2)-O(1)#12	141.66(15)
O(3)#6-Rb(1)-O(1)#8	84.68(10)	O(1)-Rb(2)-O(3)#8	84.21(12)
O(3)#7-Rb(1)-O(1)#8	103.74(9)	O(1)#12-Rb(2)-O(3)#8	103.61(10)
O(1)#2-Rb(1)-O(1)#8	152.40(18)	O(1)-Rb(2)-O(3)#2	84.21(12)
O(3)#6-Rb(1)-O(2)#9	148.26(10)	O(1)#12-Rb(2)-O(3)#2	103.61(10)
O(3)#7-Rb(1)-O(2)#9	79.99(14)	O(3)#8-Rb(2)-O(3)#2	148.53(19)
O(1)#2-Rb(1)-O(2)#9	117.91(13)	O(1)-Rb(2)-O(2)#2	126.40(12)
O(1)#8-Rb(1)-O(2)#9	64.95(13)	O(1)#12-Rb(2)-O(2)#2	85.67(14)
O(3)#6-Rb(1)-O(2)	148.26(10)	O(3)#8-Rb(2)-O(2)#2	112.69(14)
O(3)#7-Rb(1)-O(2)	79.99(14)	O(3)#2-Rb(2)-O(2)#2	54.38(14)
O(1)#2-Rb(1)-O(2)	64.95(13)	O(1)-Rb(2)-O(2)#11	126.40(12)
O(1)#8-Rb(1)-O(2)	117.91(13)	O(1)#12-Rb(2)-O(2)#11	85.67(14)
O(2)#9-Rb(1)-O(2)	54.65(16)	O(3)#8-Rb(2)-O(2)#11	54.38(14)
O(3)#6-Rb(1)-O(2)#7	73.95(13)	O(3)#2-Rb(2)-O(2)#11	112.69(14)
O(3)#7-Rb(1)-O(2)#7	50.06(14)	O(2)#2-Rb(2)-O(2)#11	60.38(16)
O(1)#2-Rb(1)-O(2)#7	123.50(12)	O(1)-Rb(2)-O(2)#14	83.80(14)
O(1)#8-Rb(1)-O(2)#7	77.38(12)	O(1)#12-Rb(2)-O(2)#14	62.76(14)
O(2)#9-Rb(1)-O(2)#7	106.14(12)	O(3)#8-Rb(2)-O(2)#14	131.64(13)

O(2)-Rb(1)-O(2)#7	129.95(11)	O(3)#2-Rb(2)-O(2)#14	75.76(13)
O(3)#6-Rb(1)-O(2)#10	73.95(13)	O(2)#2-Rb(2)-O(2)#14	112.08(13)
O(3)#7-Rb(1)-O(2)#10	50.06(14)	O(2)#11-Rb(2)-O(2)#14	148.38(12)
O(1)#2-Rb(1)-O(2)#10	77.38(12)	O(1)-Rb(2)-O(2)#13	83.80(14)
O(1)#8-Rb(1)-O(2)#10	123.50(12)	O(1)#12-Rb(2)-O(2)#13	62.76(14)
O(2)#9-Rb(1)-O(2)#10	129.95(11)	O(3)#8-Rb(2)-O(2)#13	75.76(13)
O(2)-Rb(1)-O(2)#10	106.14(12)	O(3)#2-Rb(2)-O(2)#13	131.64(13)
O(2)#7-Rb(1)-O(2)#10	46.74(14)	O(2)#2-Rb(2)-O(2)#13	148.38(12)
O(3)#6-Rb(1)-O(2)#2	62.69(12)	O(2)#11-Rb(2)-O(2)#13	112.08(13)
O(3)#7-Rb(1)-O(2)#2	154.09(7)	O(2)#14-Rb(2)-O(2)#13	56.45(15)
O(1)#2-Rb(1)-O(2)#2	50.56(10)	O(2)-V(1)-O(2)#9	109.4(3)
O(1)#8-Rb(1)-O(2)#2	102.11(12)	O(2)-V(1)-O(3)	109.5(2)
O(2)#9-Rb(1)-O(2)#2	113.00(9)	O(2)#9-V(1)-O(3)	109.5(2)
O(2)-Rb(1)-O(2)#2	89.50(8)	O(2)-V(1)-O(1)	111.84(17)
O(2)#7-Rb(1)-O(2)#2	136.38(17)	O(2)#9-V(1)-O(1)	111.84(17)
O(2)#10-Rb(1)-O(2)#2	112.26(12)	O(3)-V(1)-O(1)	104.7(3)
O(3)#6-Rb(1)-O(2)#11	62.69(12)		

Symmetry transformations used to generate equivalent atoms:

#1 -x+1,y,z	#2 -x+1/2,-y+1/2,z+1/2	#3 -x+1/2,-y+1/2,z-1/2
#4 x+1/2,y-1/2,z	#5 x+1,y,z	#6 x,y,z+1
#7 -x,-y,z+1/2	#8 -x-1/2,-y+1/2,z+1/2	#9 -x,y,z
#10 x,-y,z+1/2	#11 x-1/2,-y+1/2,z+1/2	#12 -x,-y+1,z+1/2
#13 x-1/2,y+1/2,z	#14 -x+1/2,y+1/2,z	#15 -x,-y,z-1/2
#16 -x-1/2,-y+1/2,z-1/2	#17 -x,-y+1,z-1/2	#18 x,y,z-1

Supplementary Table 3. (b) Selected bond lengths (Å) and angles (°) for CLVO.

Cs(1)-O(2)	3.043(7)	Cs(2)-O(3)	3.166(6)
Cs(1)-O(1)#1	3.114(6)	Cs(2)-O(3)#9	3.166(6)
Cs(1)-O(1)#2	3.114(6)	Cs(2)-O(3)#4	3.514(6)
Cs(1)-O(2)#3	3.119(7)	Cs(2)-O(3)#1	3.514(6)
Cs(1)-O(3)#1	3.181(6)	Li(1)-O(3)	1.884(9)
Cs(1)-O(3)#4	3.181(6)	Li(1)-O(3)#11	1.884(9)
Cs(1)-O(3)#5	3.598(6)	Li(1)-O(2)#1	2.056(14)
Cs(1)-O(3)#6	3.598(6)	Li(1)-O(1)#1	2.137(16)
Cs(2)-O(1)#7	3.061(8)	V(1)-O(3)#9	1.712(4)
Cs(2)-O(2)#2	3.086(6)	V(1)-O(3)	1.712(4)
Cs(2)-O(2)#1	3.086(6)	V(1)-O(2)	1.715(6)
Cs(2)-O(1)#8	3.145(7)	V(1)-O(1)	1.716(6)
O(2)-Cs(1)-O(1)#1	85.83(9)	O(1)#7-Cs(2)-O(3)	146.99(11)
O(2)-Cs(1)-O(1)#2	85.83(9)	O(2)#2-Cs(2)-O(3)	114.90(13)
O(1)#1-Cs(1)-O(1)#2	148.96(19)	O(2)#1-Cs(2)-O(3)	63.62(13)
O(2)-Cs(1)-O(2)#3	137.07(13)	O(1)#8-Cs(2)-O(3)	84.62(14)
O(1)#1-Cs(1)-O(2)#3	103.21(10)	O(1)#7-Cs(2)-O(3)#9	146.99(11)
O(1)#2-Cs(1)-O(2)#3	103.21(10)	O(2)#2-Cs(2)-O(3)#9	63.62(13)
O(2)-Cs(1)-O(3)#1	126.22(14)	O(2)#1-Cs(2)-O(3)#9	114.90(13)
O(1)#1-Cs(1)-O(3)#1	52.96(13)	O(1)#8-Cs(2)-O(3)#9	84.62(14)
O(1)#2-Cs(1)-O(3)#1	111.25(14)	O(3)-Cs(2)-O(3)#9	52.83(18)
O(2)#3-Cs(1)-O(3)#1	89.90(15)	O(1)#7-Cs(2)-O(3)#4	61.55(11)
O(2)-Cs(1)-O(3)#4	126.22(14)	O(2)#2-Cs(2)-O(3)#4	50.13(11)
O(1)#1-Cs(1)-O(3)#4	111.25(14)	O(2)#1-Cs(2)-O(3)#4	103.91(14)
O(1)#2-Cs(1)-O(3)#4	52.96(13)	O(1)#8-Cs(2)-O(3)#4	152.50(9)
O(2)#3-Cs(1)-O(3)#4	89.90(15)	O(3)-Cs(2)-O(3)#4	111.35(10)
O(3)#1-Cs(1)-O(3)#4	60.08(19)	O(3)#9-Cs(2)-O(3)#4	87.76(11)
O(2)-Cs(1)-O(3)#5	83.72(15)	O(1)#7-Cs(2)-O(3)#1	61.55(11)
O(1)#1-Cs(1)-O(3)#5	130.36(11)	O(2)#2-Cs(2)-O(3)#1	103.91(14)
O(1)#2-Cs(1)-O(3)#5	78.16(14)	O(2)#1-Cs(2)-O(3)#1	50.13(11)
O(2)#3-Cs(1)-O(3)#5	58.26(15)	O(1)#8-Cs(2)-O(3)#1	152.50(9)
O(3)#1-Cs(1)-O(3)#5	148.14(9)	O(3)-Cs(2)-O(3)#1	87.76(11)
O(3)#4-Cs(1)-O(3)#5	113.97(15)	O(3)#9-Cs(2)-O(3)#1	111.35(10)
O(2)-Cs(1)-O(3)#6	83.72(15)	O(3)#4-Cs(2)-O(3)#1	53.89(17)
O(1)#1-Cs(1)-O(3)#6	78.16(14)	O(3)-Li(1)-O(3)#11	115.4(8)
O(1)#2-Cs(1)-O(3)#6	130.36(11)	O(3)-Li(1)-O(2)#1	113.5(4)
O(2)#3-Cs(1)-O(3)#6	58.26(15)	O(3)#11-Li(1)-O(2)#1	113.5(4)
O(3)#1-Cs(1)-O(3)#6	113.97(15)	O(3)-Li(1)-O(1)#1	114.6(5)

O(3)#4-Cs(1)-O(3)#6	148.14(9)	O(3)#11-Li(1)-O(1)#1	114.6(5)
O(3)#5-Cs(1)-O(3)#6	52.53(17)	O(2)#1-Li(1)-O(1)#1	80.4(5)
O(1)#7-Cs(2)-O(2)#2	86.02(9)	O(3)#9-V(1)-O(3)	110.7(3)
O(1)#7-Cs(2)-O(2)#1	86.02(9)	O(3)#9-V(1)-O(2)	110.88(17)
O(2)#2-Cs(2)-O(2)#1	153.10(17)	O(3)-V(1)-O(2)	110.88(17)
O(1)#7-Cs(2)-O(1)#8	116.57(12)	O(3)#9-V(1)-O(1)	109.97(18)
O(2)#2-Cs(2)-O(1)#8	103.28(9)	O(3)-V(1)-O(1)	109.97(18)
O(2)#1-Cs(2)-O(1)#8	103.28(9)	O(2)-V(1)-O(1)	104.2(3)

Symmetry transformations used to generate equivalent atoms:

#1 -x+1/2,-y+1/2,z+1/2	#2 -x-1/2,-y+1/2,z+1/2	#3 -x,-y,z+1/2
#4 x-1/2,-y+1/2,z+1/2	#5 x-1/2,y-1/2,z	#6 -x+1/2, y-1/2, z
#7 x,y,z+1	#8 -x,-y+1,z+1/2	#9 -x, y, z
#10 x-1,y,z	#11 -x+1,y,z	#12 -x+1/2,-y+1/2,z-1/2
#13 x+1/2,y+1/2,z	#14 x+1,y,z	#15 -x,-y+1,z-1/2
#16 -x-1/2,-y+1/2,z-1/2	#17 x,y,z-1	#18 -x,-y,z-1/2

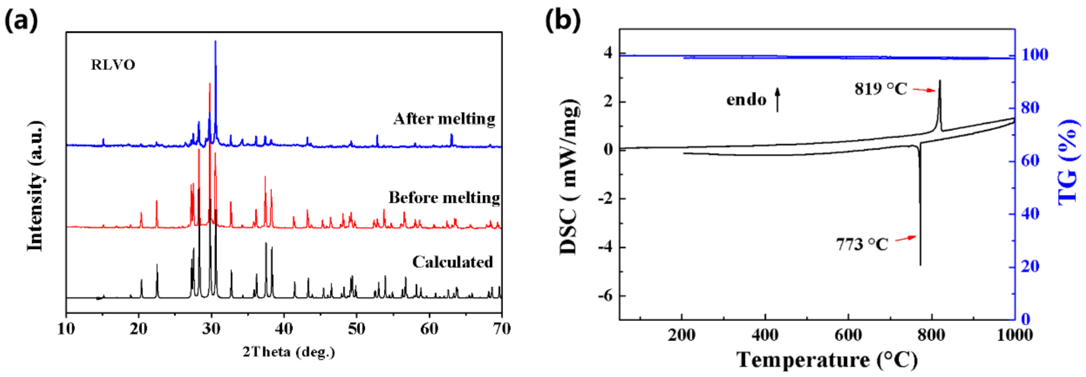

Supplementary Figure 1. (a) Calculated and experimental PXRD patterns for RLVO (b) The TG-DSC data for RLVO. Note that there is only one endothermic peak on the heating curve and one exothermic peak on the cooling curve of DSC and no obvious weight loss on the TGA curve.

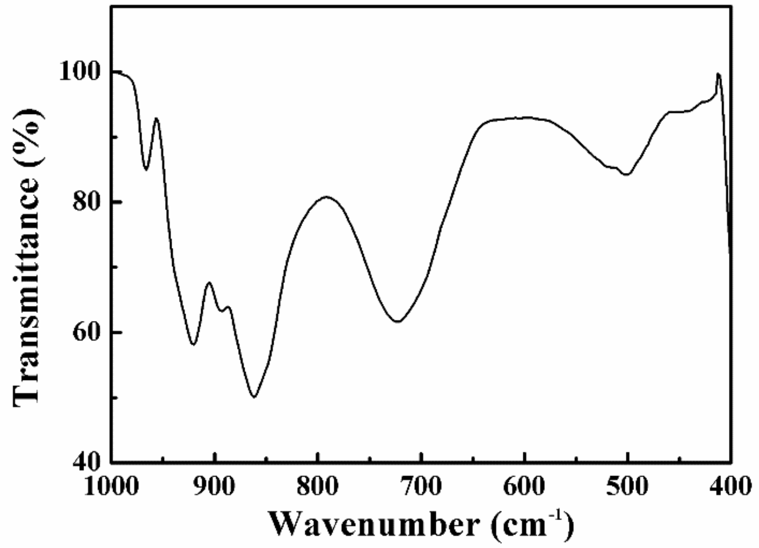

Supplementary Figure 2. (a) The IR spectrum of as-synthesized RLVO.

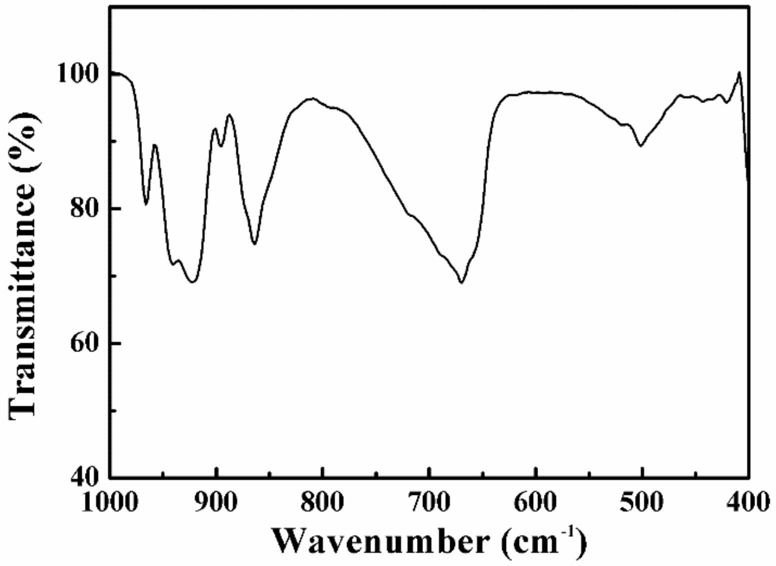

Supplementary Figure2. (b) The IR spectrum of as-synthesized CLVO.

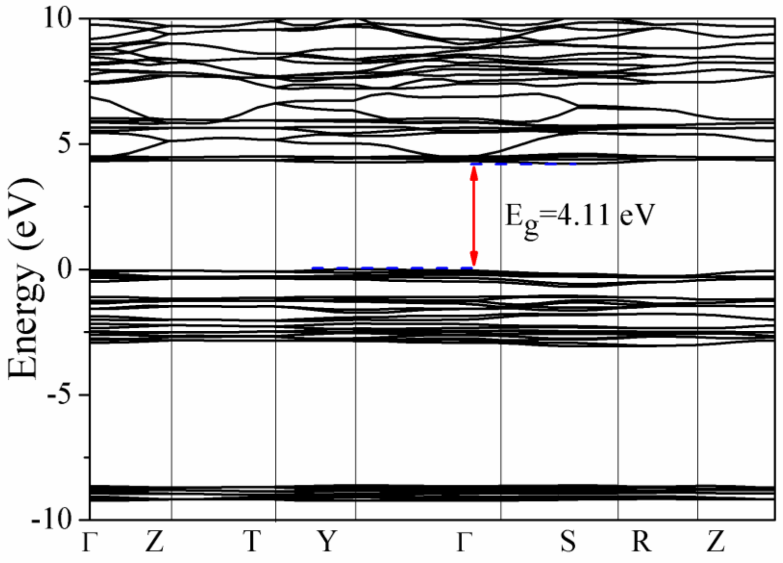

Supplementary Figure 3. (a) Electron band structure of RLVO

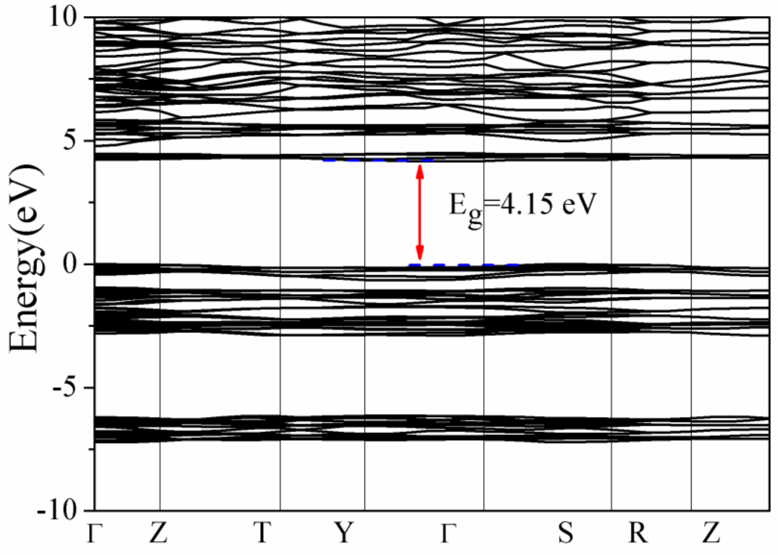

Supplementary Figure 3. (b) Electron band structure of CLVO

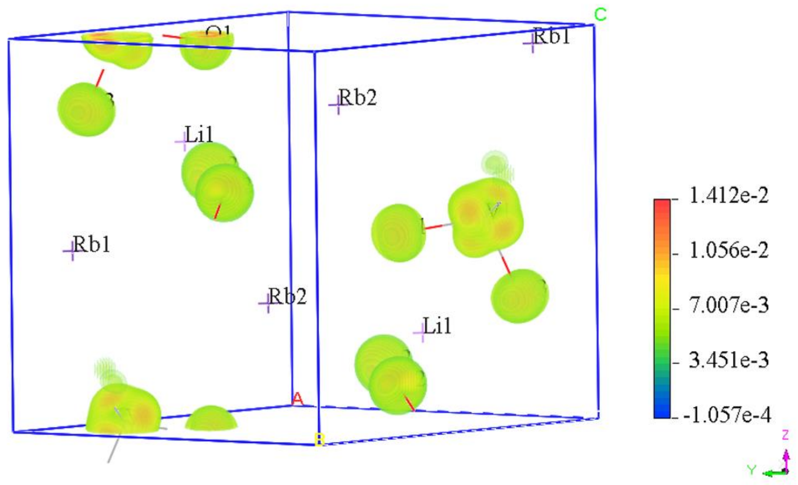

Supplementary Figure 4. The SHG-density of the virtual-electron process of the largest SHG tensors of RLVO

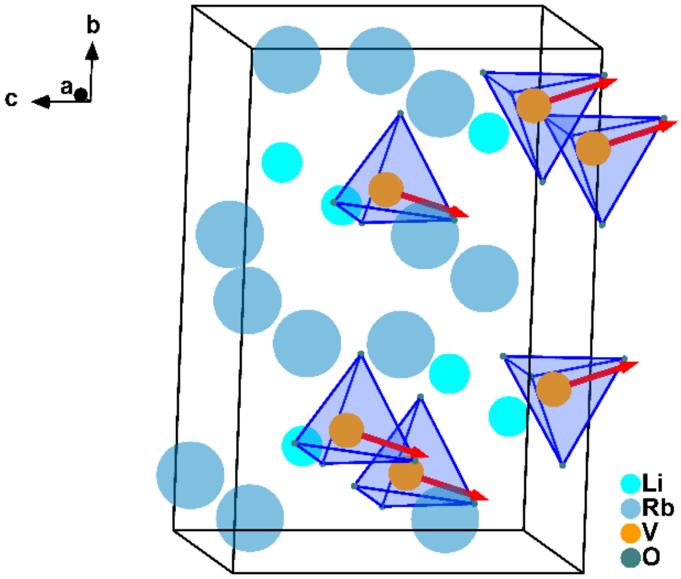

Supplementary Figure 5. The direction of the dipole moments for the VO_4 tetrahedra in the unit cell of RLVO. (The red arrows indicate the approximate directions of the dipole moments.)

Supplementary Table 4. (a) Calculation of the dipole moments for the VO_4 tetrahedra in the unit cell of RLVO and CLVO.

Crystal	Polar Unit	*x*	*y*	*z*	Magnitude	
					Total (Debye)	10^{-4} esu·cm/A^3
RLVO	VO_4	0	-0.327	-1.506	1.541	115.134
		0	0.327	-1.506	1.541	115.134
		0	0.327	-1.506	1.541	115.134
		0	0.327	-1.506	1.541	115.134
		0	-0.327	-1.506	1.541	115.134
		0	-0.327	-1.506	1.541	115.134
	Total	0	0	-9.038	9.038	168.781
CLVO	VO_4	0	-0.420	-1.653	1.705	113.671
		0	-0.420	-1.653	1.705	113.671
		0	-0.420	-1.653	1.705	113.671
		0	0.420	-1.653	1.705	113.671
		0	0.420	-1.653	1.705	113.671
		0	0.420	-1.653	1.705	113.671
	Total	0	0	-9.915	9.915	165.255

Supplementary Table 4. (b) Calculation of the dipole moments in the unit cell of RLVO and CLVO.

Crystal	Polar Unit	*x*	*y*	*z*	Magnitude	
					Total (Debye)	10^{-4} esu·cm/A^3
RLVO	LiO_4	0	0	23.670	23.670	442.019
	VO_4	0	0	-9.038	9.038	168.781
	$Rb1O_{10}$	0	0	9.842	9.842	183.787
	$Rb2O_8$	0	0	5.219	5.219	97.457
	Net Dipole Moments	0	0	19.795	19.795	369.655
CLVO	LiO_4	0	0	22.212	22.212	370.197
	VO_4	0	0	-9.915	9.915	165.255
	$Cs1O_8$	0	0	7.176	7.176	119.598
	$Cs2O_8$	0	0	-6.188	6.188	103.134
	Net Dipole Moments	0	0	8.857	8.857	147.617
